# A Tongue Lesion as a Sign of a Systemic Disease

**DOI:** 10.1155/2016/6723575

**Published:** 2016-03-22

**Authors:** Chrysoula I. Liakou, Joan Koh, Antonios Tsimpidakis, Katrina Rios, Charalabos Paskalis, Athanasios Pipilis, Dimitrios Kantianis, Thomas Georgiadis, Evangelia Razis

**Affiliations:** ^1^Third Department of Medical Oncology, Hygeia Hospital, 15123 Athens, Greece; ^2^Johns Hopkins University Internship Program, Baltimore, MD 21218, USA; ^3^Intensive Care Unit, Hygeia Hospital, 15123 Athens, Greece; ^4^First Department of Cardiology, Hygeia Hospital, 15123 Athens, Greece; ^5^Department of Otolaryngology, Hygeia Hospital, 15123 Athens, Greece; ^6^Department of Pathology, Hygeia Hospital, 15123 Athens, Greece

## Abstract

Amyloidosis is the extracellular fibril deposition of a variety of proteins, many of which circulate as plasma ingredients. It is a disease difficult to identify due to its nonspecific symptoms and manifestations. Amyloidosis of the tongue, either isolated or part of the systemic disease, is rare and its features resemble those of a tumor. We report the case of a patient with amyloidosis who presented with a tongue lesion, weakness, nonspecific arthritis, and dyspnea on exertion that resulted in multiorgan system failure.

## 1. Introduction

In western countries, incidence of AL amyloidosis is approximately 9 cases out of a million inhabitants per year, and AL amyloidosis occurs slightly more in men than in women [1]. Amyloidosis is classified on the basis of the precursor protein forming the fibril deposits (AL: primary or AA: secondary) and the distribution of amyloid deposition (localized or systemic) [2].

Types of amyloidosis seen in tertiary referral centers and inpatient medical services are AL and AA amyloidosis, although other types of amyloid are also clinically important, such as dialysis-related amyloidosis, heritable amyloidosis, age-related systemic amyloidosis, and organ-specific amyloid. AL amyloidosis is the most common form of systemic amyloidosis and can respond to chemotherapy directed at the underlying plasma-cell dyscrasia. Common sites of AL amyloidosis are spleen, adrenal glands, liver, and gut. Amyloid deposit in the tongue is uncommon, accounting for less than 9% of all types of amyloidosis [3]. We report a case of amyloidosis of the tongue that was diagnosed postmortem in a patient who died of multisystem organ failure.

## 2. Case Report

A seventy-seven-year-old female with history of hypertension, gastroesophageal reflux, hypercholesterolemia, and an episode of pericarditis at the age of thirty-five was admitted to the hospital in February 2014 for significant dyspnea, lower extremity edema, and a painful tongue lesion.

The patient described gradual deterioration of her general health over months: generalized fatigue, inability to perform daily activities, lower extremity edema, and transient red bullae on the lips that subsided on their own.

Her primary care physician referred her to a rheumatologist where she was diagnosed with nonspecific arthritis. Electromyogram revealed bilateral carpal tunnel syndrome and right sided injury to the ulnar nerve. Laboratory evaluation was significant for positive antinuclear antibodies (ANA) and anti-double stranded DNA antibodies (anti-ds-DNA). Based on a tentative diagnosis of systemic lupus erythematosus, the patient started hydroxychloroquine and prednisolone. During the monthly follow-up visit, the patient had anemia, and she was referred to a hematologist. At the same time, she became increasingly unable to eat because of a lesion on the left side of her tongue.

She was thus brought to the emergency room and the tongue lesion was evaluated by an otolaryngologist, who found her to be debilitated and in distress. The patient was admitted to the hospital by the medical oncology team since the tongue lesion was thought to be cancerous. On admission, the patient was an obese, ill-looking woman with shortness of breath on minimal exertion. Lung auscultation revealed bilateral wet rhonchi and she was tachycardic with bilateral pitting edema on lower extremities. Her tongue appeared beefy red. There was no cervical lymphadenopathy.

The patient was treated initially with oxygen, diuretics, inhalers, and a blood transfusion. Her general condition remained poor and unstable, with increasing need for respiratory support. The next morning she deteriorated to the point where she suffered a cardiac arrest while being transferred to the ENT suite for a tongue biopsy.

**Figure 1:**
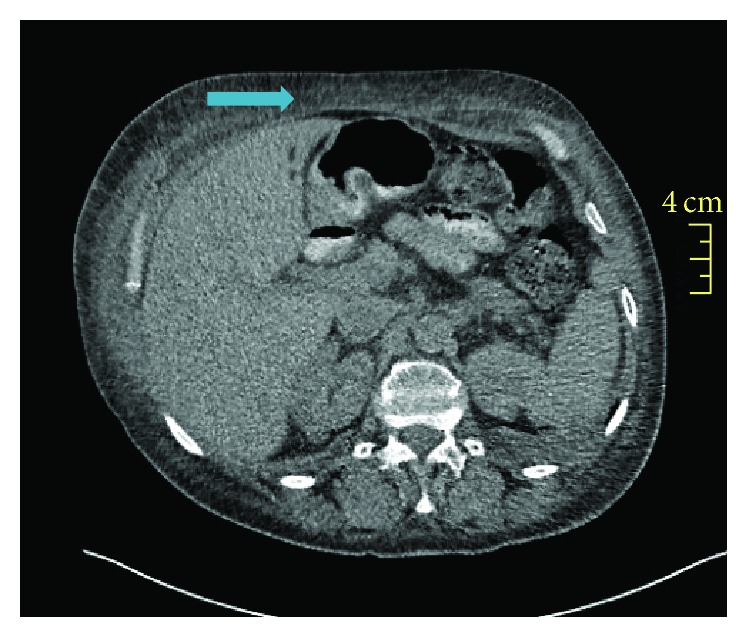
77-year-old female patient with a tongue lesion and heart failure. Computed tomography of the abdomen reveals fat opacification (bold arrow).

After resuscitation, she was transferred to the intensive care unit. Computed tomography of the chest and abdomen showed bilateral, well-defined, dense pulmonary infiltrates and diffuse opacification of the abdominal subcutaneous fat (Figure 1). Echocardiogram showed global hypokinesis, most prominent in the anterior wall; B-type natriuretic peptide (BNP) was elevated while creatinine kinase MB (CKMB) and troponin enzymes were borderline. Coronary catheterization was negative for myocardial ischemia or coronary stenosis. Her disease course was complicated by takotsubo cardiomyopathy with a pathognomonic echocardiogram of hypokinetic left ventricle wall except for the base. The patient underwent biopsy of the tongue lesion while being intubated in the unit.

In the ICU, she required high doses of vasopressors and antiarrhythmic agents but soon developed adult respiratory distress syndrome (ARDS) and renal dysfunction. She eventually succumbed in the ICU from multiorgan system failure.

**Figure 2:**
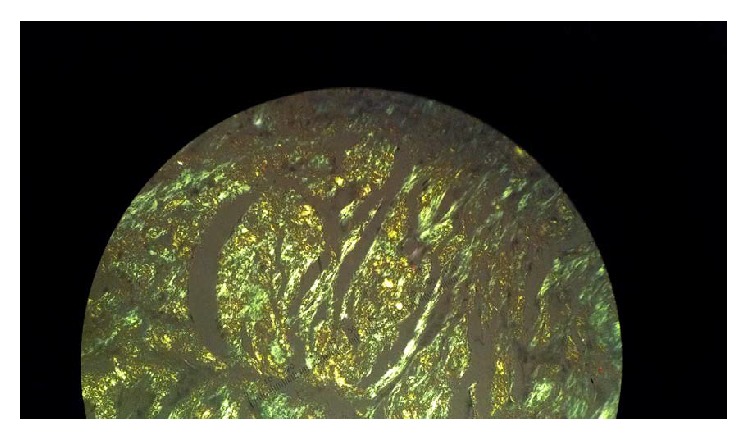
77-year-old female patient with a tongue lesion and heart failure. Tongue biopsy. A Congo red stain viewed under polarization shows the characteristic apple-green birefringence of amyloid.

The biopsy of the tongue yielded amyloidosis (Figure 2). In particular, the lesion exhibited homogenous eosinophilic amyloid-like material. Special staining with Congo red showed amyloid material as peach-red color under light microscopy with apple-green birefringence under polarized light.

## 3. Discussion

Systemic amyloidosis is difficult to identify due to its nonspecific symptoms, wide range of manifestations, and rarity. Clinically, fatigue and weight loss are commonly the initial symptoms. Other various symptoms may develop depending on the extent and location of the amyloid deposition; for AL amyloidosis, symptoms include dyspnea, macroglossia, unexplained heart failure, bruising around the eye, loss of appetite, nephrotic syndrome, peripheral neuropathy, and carpal tunnel syndrome. Frequently, the diagnosis of amyloidosis is not made until signs appear in organs known to be commonly involved such as the heart and kidney, which are affected in 60% and 70%, respectively, in patients with the disease [4, 5].

The patient in this case report had symptoms consistent with AL amyloidosis: lower extremity edema, dyspnea, tongue lesion, bilateral carpal tunnel syndrome, multiple cardiac complications, and renal dysfunction. She also had marked haziness of the abdominal wall fat on CT (Figure 1), a common source of diagnostic biopsy material. AL amyloidosis diagnosis requires (1) demonstration of amyloid in tissue and (2) demonstration of plasma cell dyscrasia. Amyloid deposits demonstrate apple-green birefringence when stained with Congo red and viewed under polarizing microscopy. Fine-needle aspiration of abdominal fat is a simple procedure and is positive in 70% of patients [6]. Once a tissue diagnosis has been established, confirmation of AL disease requires bone marrow biopsy showing predominance of *κ*- or *λ*-producing plasma cells or the presence of monoclonal light chain in the serum or urine. Immunofixation electrophoresis should be performed in the serum and urine since the concentration of the monoclonal light chain often is too low to be detected by simple protein electrophoresis. Serum free-light-chain (FLC) assay, a nephelometric immunoassay, has a sensitivity for circulating free light chains that is reportedly 10-fold that of immunofixation electrophoresis. The FLC assay is quantitative and its utility is not only in making the diagnosis but also in assessing progression and response to treatment [7, 8].

Amyloidosis of the tongue is uncommon and its features resemble a tumor. In 19% of 141 biopsy proven cases of amyloidosis, the head and neck area was affected and the tongue was the most frequent site. Differential diagnosis of a tongue lesion and systemic disease is not very extensive and it includes infectious diseases such as herpes simplex virus, syphilis, and coxsackie virus, but the patient never had any type of rash or history of fever and exposure history. Moreover, her condition should have deteriorated more rapidly, in the case disseminated infection, not over years [9].

Oral cancer, especially metastatic, such as squamous cell carcinoma, lymphoma, Kaposi's sarcoma, melanoma, and rhabdomyosarcoma may present as red beefy tongue abnormalities and systemic disease. Rheumatologic diseases rarely present with a tongue lesion. Metabolic disorders such as scurvy and iron deficiency anemia (Plummer-Vinson syndrome) may cause tongue abrasion and generalized malaise but the patient was not a malnourished individual in order to consider that type of disease [9].

MRI or CT is the most commonly used modality for assessment of tongue lesions, and biopsy remains the diagnostic gold standard. The initial diagnosis assumption in this case report was some type of neoplasia, and that is the reason the oncology team was consulted upon arrival in the ER. The patient had enhanced abdominal fat on CT scan of the abdomen which could alert the team of that diagnosis; however, the disease was long neglected and rapidly progressive during hospitalization, so that the patient succumbed to it [10].

Overall, amyloidosis carries a poor prognosis. When left untreated, median survival estimates are 12 to 18 months. The aim of AL amyloidosis therapy is to rapidly reduce the amyloidogenic monoclonal light chain load, by controlling the underlying plasma cell dyscrasia [11].

Once a patient develops systemic disease, heart and multiorgan failure, amyloidosis should be on the differential diagnosis. The earlier the diagnosis is made, the faster the therapies can begin, delaying or averting the development of irreversible conditions such as cardiomyopathy [12].

## 4. Conclusion

Amyloidosis is a rare group of diseases and often difficult to recognize at the time of diagnosis. We present this case in order to alert the medical community to this insidiously presenting disease, which can masquerade as tongue cancer and heart failure.
